# Comparison of antimicrobial prescribing for dental and oral infections in England and Scotland with Norway and Sweden and their relative contribution to national consumption 2010–2016

**DOI:** 10.1186/s12903-020-01163-x

**Published:** 2020-06-16

**Authors:** Andrew Smith, Rania Al-Mahdi, William Malcolm, Nikolaus Palmer, Gunnar Dahlen, Mohammed Al-Haroni

**Affiliations:** 1 University of Glasgow, Glasgow, Scotland; 2 Department of Clinical Dentistry, Faculty of Health Sciences, UiT the Arctic University of Norway, Tromso, Norway; 3 Health Protection Scotland, NHS National Services Scotland, Glasgow, Scotland, UK; 4 Health Education England-North West, Liverpool, England, UK; 5 Institute of odontology, University of Gothenburg, Gothenburg, Sweden; 6 Centre for New Antimicrobial Strategies, UiT the Arctic University of Norway, Tromso, Norway

**Keywords:** Dentistry, Prescriptions, Antibiotics, Consumption, Defined daily doses (DDD), Phenoxymethyl penicillin, Amoxicillin, Metronidazole, Clindamycin, Spiramycin

## Abstract

**Background:**

Prescribing in dental practice has a relatively small but important contribution to the quantity of antibiotics prescribed in primary care. This study aimed to analyse antibiotic prescribing in dentistry over time (2010–2016) in 4 different Northern European countries and their relative contribution to national outpatients consumption.

**Methods:**

This retrospective study evaluated the frequency and number of national antibiotic prescriptions written by dentists in England, Scotland, Norway and Sweden. The consumption of such antibiotics was measured using WHO defined daily doses (DDDs), DDDs per 100,000 inhabitants per day (DIDs_100,000_).

**Results:**

A total of more than 27 million prescriptions (27,026,599) archived between 2010 and 2016 from the four countries were analysed. The national contribution of Norwegian dentists to the total primary care prescription during this period was 8%. The corresponding figures for Sweden, Scotland and England were 7, 6, and 8%. Dental contribution to National antibiotic use in all four countries has decreased over the study time period for commonly prescribed antibiotics in dentistry, i.e., the beta-lactams (Phenoxymethyl penicillin/Amoxicillin) and metronidazole. There were less numbers of prescriptions by dentists in Norway and Sweden compared to England and Scotland. Marked differences in some classes of antibiotics were noted with Phenoxymethyl penicillin dominating in Sweden/Norway compared to Amoxicillin and Metronidazole in England/Scotland. In England and Scotland, dentists were the largest prescribers of metronidazole in primary care. Clindamycin prescriptions was higher in Norway and Sweden.

**Conclusion:**

Noticeable differences exist in prescribing patterns for the management of oral infections. High levels of metronidazole use in England and Scotland also require further analysis. All countries over the study period showed a decrease in total numbers of antibiotics prescribed.

## Background

Prescribing in dental practice has a relatively small but important contribution to the quantity of antibiotics prescribed in primary care. Dentists prescribe antibiotics to treat acute bacterial intra-oral infections and some cases of chronic periodontitis. The majority of acute dento-alveolar infections should be managed primarily by surgical or local measures to control the source of infection (extraction, root canal therapy or incision and drainage) with professional guidelines re-enforcing this basic tenet of infection source control in dental practice [1–3]. However, there is evidence that there has been both inappropriate qualitative and quantitative prescribing for dental infections [4–9].

The trend of antibiotic prescriptions over time is influenced by many factors, such as disease levels, access to dental services, prescribing guidelines, patients’ attitude toward antibiotic prescribing, and incidence of antimicrobial resistance (AMR) [6, 10]. The prevalence of untreated dental caries and severe periodontal diseases, which could lead to dento-alveolar infections can be described in terms of Years Lived with Disability (YLD) standardised rate per capita, which was highest in Scotland (0.579), followed by England (0.51), Norway (0.481) and then Sweden (0.473) [11]. Accurate estimations of AMR in bacterial populations isolated from acute dental infections are difficult to interpret. Review articles have reported resistance rates to vary between 9 and 54% [12]. The AMR data is confounded by changes in bacterial taxonomy, methods of susceptibility testing and breakpoints used. With increasing trends of antibiotic resistant infections across many bacterial species and clinical specialties, adoption of antimicrobial stewardship principles by all prescribers is vital. In addition, it is timely to remember that dental antibiotic use contributes to the selection pressure for development of AMR generally and not just linked to dental infections or oral pathogens [13–15]. An important principle of antimicrobial stewardship is surveillance of antibiotic use and feedback to prescribers to drive quality improvement in the empirical use of antibiotics [16]. National data on antibiotic use by dentists can be used to identify priorities for antibiotic stewardship in dentistry and inform management and prescribing guidance. There are some reports of good outcomes following a variety of interventions [17].

The objective of this study was to compare the national use of antibiotics by dental prescribers in England and Scotland with Norway and Sweden. Report their relative contribution to the total national antibiotic consumption by outpatients, and review differences in prescribing patterns to ascertain whether lessons can be used to inform antimicrobial stewardships policies in the four countries.

## Methods

### Data source

For the study period 2010–2016, except for England where data was available from 2011, the data on National Health Service (NHS) antibiotic prescriptions written by dentists dispensed in the community was obtained from the NHS Business Service Authority for England and from the Prescribing Information System, a database of all NHS prescriptions dispensed in Scotland held by NHS National Services Scotland. The data on dental antibiotic use in Norway, was obtained from the Norwegian prescription database (NorPD) [18], while the dental antibiotic use from Sweden was obtained from the Public Health Agency of Sweden. The aggregated data from the four countries also include the number of dentists practicing in NHS England and NHS Scotland and all dentists practicing in Norway and Sweden. Obtaining of the anonymous aggregated data on antibiotic use by dentists was approved by the respective authorities in each country.

### Classification of data

The data obtained on antibiotic use was classified according to the anatomical therapeutic chemical (ATC) classification system, using the WHO defined daily dose (DDD) for each drug [19]. In this system, antibiotics for systemic use fall into ATC group “J01” except for metronidazole where it falls into ATC group “P01”. The data included the number of antibiotic prescription items written by dentists dispensed in the community to each antibiotic, total national antibiotic use expressed as DDD in the country in each year and the DDD prescribed by dentists for each respective year. Antibiotic use by dentists as a proportion of total antibiotic use in the community was calculated.

### Antibiotic groups

In the current study, all dental prescriptions dispensed in the community were analysed. In dentistry, the use of antibiotics to treat dental and oral infections usually relies on five groups of antibiotics. These are beta-lactams (primarily Phenoxymethyl penicillin and amoxicillin), macrolides, lincosamides, tetracyclines, and metronidazole. Beta-lactams: Amoxicillin, ATC code J01CA04; Phenoxymethyl penicillin (penicillin V), ATC code J01CE02; Co-amoxiclav (Augmentin), J01CR02, Cefalexin, ATC codes J01DB01 and Cefradine, ATC code J01DB09. Macrolides and Lincosamides: Erythromycin, ATC code J01FA01; Spiramycin, ATC code J01FA02; Clarithromycin, ATC code J01FA09; Azithromycin, ATC code J01FA10; Clindamycin, ATC code J01FF01. Tetracyclines: Doxycycline, ATC code J01AA02; Oxytetracycline, ATC code J01AA06 and Tetracycline, ATC code J01AA07. Metronidazole: Metronidazole, ATC code P01AB01.

### Comparison of antibiotic consumptions attributed to dental prescription between England, Scotland, Norway and Sweden

Trends in the consumption of antibiotics were assessed for the study period (2010–2016). The proportion of each antibiotic prescribed compared to the total antibiotic prescription by dentists was calculated. The proportion of antibiotic consumption attributed to dentistry-based prescription to the total national primary care consumption was also calculated. The mid-year populations in England and Scotland for each year were extracted from the office of National Statistics. The population size in Norway and Sweden for each year was obtained from the Statistical Bureau in Norway and Sweden. To better illustrate the consumption of antibiotics by dental prescriptions in the four countries, the data was calculated for each antibiotic prescribed by dentists in each country and expressed as a number of DDD per 100,000 inhabitants per day (DID_100,000_). The average number of antibiotic prescriptions dispensed in the community per dentist per year in each country was calculated. The proportion (%) of each antibiotic to the total antibiotics dispensed in the community was also calculated.

## Results

**Fig. 1:**
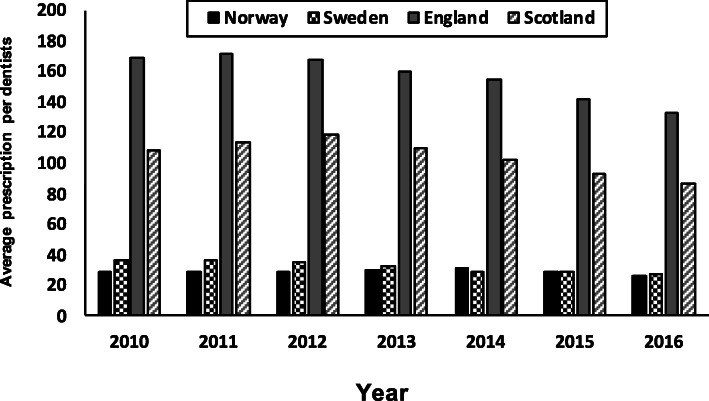
Average number of antibiotic prescriptions dispensed in the community per dentist per year in Norway, Sweden, England and Scotland

A total of 21,719,095 dental prescriptions were dispensed in England, 2,591,077 Scotland, 1,048,568 Norway and 1, 717,859 were in Sweden, during the study period. The number of prescriptions issued by dentists per year in England, Scotland, Norway and Sweden are listed in Table 1 and the average prescription per dentist per year in Norway and Sweden versus England and Scotland is presented in Fig. 1.

**Table 1:** Total number of prescriptions for antibiotics dispensed in the community by dentists (Prescriptions), total number of dentists (dentists) and average prescription by dentists (average prescription per dentist)

**Year**	**England**	**Scotland**	**Norway**	**Sweden**
No. of prescriptions/No. of dentists (Average prescription per dentists)				
2010	3,850,773/22799 (169)	367,268/3396 (108)	146,672/5121 (29)	268,539/7509 (36)
2011	3,913,551/22920 (171)	395,238/3466 (114)	149,161/5183 (29)	272,291/7630 (36)
2012	3,907,934/23201 (168)	411,207/3454 (119)	152,027/5223 (29)	266,824/7710 (35)
2013	3,790,869/23723 (160)	394,998/3602 (110)	153,993/5134 (30)	246,454/7731 (32)
2014	3,682,513/23947 (154)	369,066/3604 (102)	155,644/5102 (30)	225,820/7777 (29)
2015	3,425,450/24089 (142)	335,136/3610 (93)	150,827/5256 (30)	221,383/7699 (29)
2016	3,190,045/ 24,007 (133)	318,164/3670 (87)	140,244/5334 (26)	216,548/7700 (28)
Mean ± SD (95%CI)				
	156.71 ± 14.60 (156.71 ± 10.81)	104.71 ± 11.45 (104.71 ± 8.48)	29 ± 1.41 (29 ± 1.048)	32.14 ± 3.53 (32.14 ± 2.61)

**Fig. 2:**
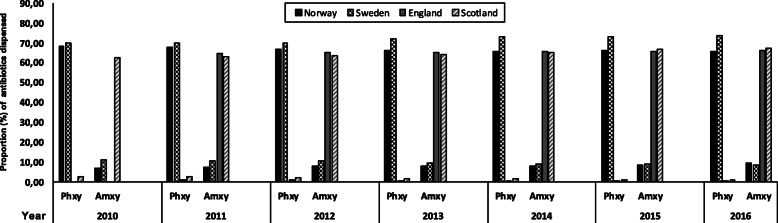
The proportion (%) of Phenoxymethyl penicillin and Amoxicillin dispensed in Norway, Sweden, England and Scotland to the total antibiotics dispensed by the general dental practitioners. Data from England is available from 2011 to 2016

The highest annual rate of antibiotic prescribing per dentist was in England in 2011 (*n* = 171), by 2016 this had declined to 133 antibiotic prescriptions per year (Fig. 1), this represented the highest number of prescriptions per dentist from all four countries. In Scotland the prescription rate per dentist peaked in 2012 (*n* = 119) and had declined to 87 per annum by 2016. Norway had the lowest rates of prescriptions per dentist (peaking at 31 in 2014) with a decline in prescriptions to 26 per year by 2016. Swedish prescribing levels were highest in 2010 (*n* = 36) and declined to 28 per annum by 2016. The dental contribution to the total of antibiotics prescribed in the community for each Country over the period 2010–16 was 8, 6, 8, and 7% for England, Scotland, Norway and Sweden. The proportion (%) of Phenoxymethyl penicillin and Amoxicillin dispensed in Norway and Sweden versus England and Scotland by dentists to the total antibiotics dispensed in the community by general dental practitioners is presented in Fig. 2.

**Fig. 3:**
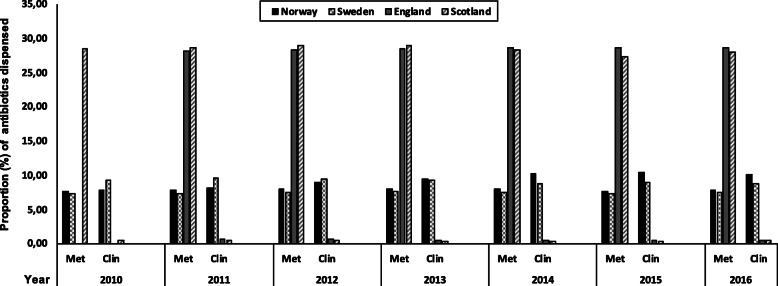
The proportion (%) of Metronidazole and Clindamycin dispensed in Norway, Sweden, England and Scotland to the total antibiotics dispensed by the general dental practitioners. Data from England is available from 2011 to 2016

The proportion (%) of Metronidazole and Clindamycin dispensed in Norway and Sweden versus England and Scotland to the total antibiotics dispensed by general dental practitioners is presented in Fig. 3.

The DID_100,000_ attributed to dental prescriptions in England, Scotland, Norway and Sweden in every year from 2010 to 2016 are presented in Table 2. Table 3 presents the average DID_100,000_ attributed to dental prescriptions in the study period.

**Table 2:** Number of DDD per 100,000 inhabitants per day (DID_100,000_) attributed to dental prescriptions in England, Scotland, Norway and Sweden

**Year**	**Antibiotics**	**England**	**Scotland**	**Norway**	**Sweden**
		**DID**_**100,000**_			
2010	**(Amxy) – (Phxy) – (Met)**	No data	(70.28)–(2.02) − (10.40)	(7.57)–(68.49) – (3.52)	(10.28)–(80.37) – (3.29)
	**(Clin) – (Ery) – (Doxy)**	No data	(0.38)–(4.62) − (1.32)	(4.03)–(2.94) –(1.84)	(4.15)–(0.54) – (1.36)
	**(Tet) – (Oxy) – (Azy)**	No data	(0.12)–(0.05) − (0)	(0.56)–(0.12) – (1.06)	(0.08)–(0) – (0.02)
	**(Coamxy) – (Spir)**	No data	(0.26)–(0)	(0)–(0.17)	(0)–(0)
2011	**(Amxy) – (Phxy) – (Met)**	(89.93)–(0.94) – (13.66)	(76.39)–(1.99) – (11.35)	(7.82)–(67.70) – (3.55)	(10.26)–(80.49) – (3.26)
	**(Clin) – (Ery) – (Doxy)**	(0.45)–(6.16) – (0.52)	(0.34)–(4.67) − (1.43)	(4.21)–(2.84) – (1.83)	(4.37)–(0.49) – (1.46)
	**(Tet) – (Oxy) – (Azy)**	(0.16)–(0.06) – (0.01)	(0.14)–(0.05) − (0)	(0.43)–(0.06) – (1.20)	(0.09)–(0) – (0.02)
	**(Coamxy) – (Spir)**	(0.34)–(0)	(0.28)–(0)	(0)–(0.15)	(0)–(0)
2012	**(Amxy) – (Phxy) – (Met)**	(89.45)–(0.76) – (13.56)	(79.66)–(1.78) – (11.96)	(8.27)–(66.06) – (3.66)	(10.16)–(78.05) – (3.27)
	**(Clin) – (Ery) – (Doxy)**	(0.42)–(6.03) – (0.49)	(0.33)–(4.64) – (1.54)	(4.56)–(2.61) – (2.0)	(4.21)–(0.40) – (1.51)
	**(Tet) – (Oxy) – (Azy)**	(0.13)–(0.05) –(0.04)	(0.13)–(0.05) – (0.02)	(0.37)–(0) – (1.19)	(0.10)–(0) – (0.02)
	**(Coamxy) – (Spir)**	(0.42)–(0)	(0.20)–(0)	(0)–(0.15)	(0) – (0)
2013	**(Amxy) – (Phxy) – (Met)**	(87.14)–(0.62) – (13.21)	(78.38)–(1.48) – (11.68)	(8.88)–(65.37) – (3.78)	(8.58)–(74.74) – (3.02)
	**(Clin) – (Ery) – (Doxy)**	(0.36)–(5.73) – (0.45)	(0.29)–(4.29) – (1.24)	(4.79)–(2.53) – (1.82)	(3.73)–(0.31) – (1.27)
	**(Tet) – (Oxy) – (Azy)**	(0.11)–(0.04) – (0.05)	(0.14)–(0.04) – (0.03)	(0.34)–(0) – (1.18)	(0.07)–(0) – (0.03)
	**(Coamxy) – (Spir)**	(0.43)–(0)	(0.25)–(0)	(0)–(0.13)	(0) – (0)
2014	**(Amxy) – (Phxy) – (Met)**	(87.19)–(0.48) – (13.08)	(85.35)–(1.23) – (11.03)	(9.45)–(65.05) – (3.80)	(7.43)–(69.76) – (2.68)
	**(Clin) – (Ery) – (Doxy)**	(0.33)–(5.35) – (0.38)	(0.24)–(3.84) – (1.09)	(5.21)–(2.31) – (1.86)	(3.21)–(0.25) – (1.04)
	**(Tet) – (Oxy) – (Azy)**	(0.09)–(0.04) – (0.06)	(0–09) – (0.02) – (0.02)	(0.34)–(0) – (1.09)	(0.06)–(0) – (0.03)
	**(Coamxy) – (Spir)**	(0.46)–(0)	(0.20)–(0)	(0)–(0.14)	(0) – (0)
2015	**(Amxy) – (Phxy) – (Met)**	(85.04)–(0.37) – (12.46)	(85.90)–(0.92) – (9.92)	(9.39)–(63.66) – (3.52)	(7.37)–(68.33) – (2.51)
	**(Clin) – (Ery) – (Doxy)**	(0.30)–(4.85) – (0.35)	(0.18)–(3.37) – (1.02)	(5.27)–(1.98) – (1.81)	(3.18)–(0.24) – (0.96)
	**(Tet) – (Oxy) – (Azy)**	(0.07)–(0.03) – (0.06)	(0.06)–(0.03) – (0.02)	(0.28)–(0) – (0.95)	(0.05)–(0) – (0.03)
	**(Coamxy) – (Spir)**	(0.44)–(0)	(0.16)–(0)	(0)–(0.13)	(0) – (0)
2016	**(Amxy) – (Phxy) – (Met)**	(80.93)–(0.29) – (11.77)	(82.92)–(0.68) – (9.56)	(9.52)–(58.65) – (3.39)	(6.99)–(66.86) – (2.52)
	**(Clin) – (Ery) – (Doxy)**	(0.28)–(4.47) – (0.32)	(0.19)–(2.52) – (0.86)	(4.58)–(1.81) – (1.37)	(3.02)–(0.21) – (0.88)
	**(Tet) – (Oxy) – (Azy)**	(0.05)–(0.03) – (0.06)	(0.05)–(0.03) – (0.01)	(0.27)–(0) – (0.82)	(0.04)–(0) – (0.04)
	**(Coamxy) – (Spir)**	(0.40)–(0)	(0.13)–(0)	(0)–(0.11)	(0) – (0)

*Amxy* Amoxicillin, *Phxy* Phenoxymethyl penicillin, *Met* Metronidazole, *Clin* Clindamycin, *Ery* Erythromycin, *Doxy* Doxycycline, *Tet* Tetracycline, *Oxy* Oxytetracycline, *Azy* Azithromycin, *Coamxy* Co-amoxiclav

**Table 3:** The average DDD per 100,000 inhabitants per day (DID_100,000_) attributed to dental prescriptions during the study period England, Scotland, Norway and Sweden

**Antibiotics**	**Average of DID**_**100,000**_**dispensed in the community from 2010 to 2016**			
	**England**^**a**^	**Scotland**	**Norway**	**Sweden**
(Amxy)	86.73	79.85	8.70	8.72
(Phxy)	0.58	1.44	65.00	74.09
(Cflxn)	0.27	0.24	0	0
(Cfrdn)	0.04	0.09	0	0
(Coamxy)	0.42	0.21	0	0
(Met)	12.95	10.84	3.60	2.94
(Clin)	0.36	0.24	4.66	3.70
(Ery)	5.43	3.99	2.43	0.35
(Doxy)	0.42	1.21	1.79	1.21
(Tet)	0.10	0.10	0.37	0.07
(Oxy)	0.04	0.04	0.03	0
(Azy)	0.05	0.02	1.07	0.03
(Clary)	0.21	0.10	0.06	0.02
(Spir)	0	0	0.14	0

*Amxy* Amoxicillin, *Phxy* Phenoxymethyl penicillin, *Cflxn* Cefalexin, *Cfrdn* Cefradine, *Coamxy* Co-amoxiclav, *Met* Metronidazole, *Clin* Clindamycin, *Ery* Erythromycin, *Doxy* Doxycycline, *Tet* Tetracycline, *Oxy* Oxytetracycline, *Azy* Azithromycin, *Clary* Clarithromycin, *Spir* Spiramycin

^a^ Data from England is available from 2011 to 2016

### Analysis of each antibiotic prescribed for dental infections over time

There were marked differences in patterns of consumption of antibiotic attributed to dental prescription between England and Scotland versus Norway and Sweden. In England and Scotland, Amoxicillin, Metronidazole, Erythromycin and Phenoxymethyl penicillin (ordered from highest to lowest) were the top four antibiotics prescribed by dentists. However, in Norway and Sweden, Phenoxymethyl penicillin, Amoxicillin, Clindamycin and then Metronidazole (ordered from highest to lowest) were the top four antibiotics prescribed by dentists.

### Phenoxymethyl penicillin (penicillin V) consumption

This was the most commonly prescribed antibiotic in Norway and Sweden by dental practitioners. In line with the overall decrease in total antibiotic prescriptions between 2010 and 2016 there was a trend towards decrease in the DID_100,000_ for Phenoxymethyl penicillin. Sweden had the highest reduction (16%) in DID_100,000_ of this antibiotic declining to 66.86 in 2016 from 80.37 in 2010. This was followed by Norway (14% reduction) with a DID_100,000_ of 68.49 in 2010 declining to 58.65 in 2016. In contrast, Phenoxymethyl penicillin was one of the least commonly prescribed antibiotics by dental practitioners in England and Scotland over the study period. For England in 2011 the DID_100,000_ was 0.94 falling to 0.29 in 2016 and in Scotland a fall from 2.02 in 2010 to 0.68 in 2016.

### Amoxicillin consumption

Amoxicillin was prescribed more frequently in England and Scotland than in Norway and Sweden. The highest number of DID_100,000_ was in England in 2011 (*n* = 89.93) declining to 80.93 in 2016. In contrast the Amoxicillin DID_100,000_ in Scotland rose from 70.28 in 2010 to 82.92 in 2016. Sweden saw a fall in DID_100,000_ from 10.28 to 6.99 in 2016. Conversely, Norway saw an increase in the DID_100,000_ between 2010 (*n* = 7.57) rising to a DID_100,000_ of 9.52 in 2016.

### Metronidazole consumption

For England and Scotland Metronidazole was the second most frequently prescribed antibiotic, with a DID_100,000_ in 2011 of 13.66 for England and in Scotland peaking at a DID_100,000_ of 11.96 in 2012 and falling to 9.56 in 2016. Both England and Scotland prescribed more Metronidazole than Norway and Sweden. In Norway and Sweden, this was the fourth most frequently prescribed antibiotic (after Phenoxymethyl penicillin, Amoxicillin and Clindamycin). The Metronidazole DID_100,000_ for Sweden and Norway both showed deceases over the 2010 and 2016 period with Sweden showing a decline in DID_100,000_ of 3.29 in 2010 to 2.52 in 2016 and Norway’s DID_100,000_ decreasing from 3.52 in 2010 to 3.39 in 2016.

### Erythromycin consumption

In England and Scotland this was the third most commonly prescribed antibiotic but the fifth most commonly prescribed antibiotic in Norway and Sweden (after Phenoxymethyl penicillin, amoxicillin, clindamycin and metronidaxole). All countries showed a decline in erythromycin prescriptions over the study period with the largest DID_100,000_ in England (*n* = 6.16) in 2011 and the lowest DID_100,000_ in Sweden in 2016 of 0.21.

### Clindamycin consumption

Norway and Sweden had the highest DID_100,000_ consumption for Clindamycin, with a DID_100,000_ of 4.03 in 2010 for Norway rising to 4.58 in 2016 and from a DID_100,000_ of 4.15 in 2010 to 3.02 in 2016 for Sweden. In contrast the Clindamycin consumption were lower for England and Scotland varying from a 0.45 DID_100,000_ in England in 2011 to 0.19 DID_100,000_ in 2016 for Scotland.

### Other antibiotics consumption

The Tetracycline class of antibiotics were prescribed at approximately the same DID_100,000_ levels across the four countries. In contrast, for Norway the DID_100,000_ for Azithromycin (0.8 in 2016) and Spiramycin (0.1 in 2016) was higher than in the other three countries. The DID_100,000_ for Augmentin (Co-amoxiclav) prescriptions was significantly higher in England and Scotland in 2016 running at 0.4 and 0.13, respectively.

## Discussion

The Global Burden of Disease Study 2016 estimated that oral diseases affected half of the world’s population [20]. Untreated dental decay, which could lead to dento-alveloar infections, in permanent teeth affects 2.3 billion people, and untreated dental decay in primary teeth affects more than 560 million children worldwide. Severe periodontal diseases that may result in tooth loss and affect general health and wellbeing, was estimated to be the 11th most prevalent disease globally [20]. Dentists prescribe a panel of antibiotics to treat and manage acute dento-alveloar infections. Differences in total volumes of antibiotics prescribed as well as differences in prescription pattern in different geographical locations to treat and manage dento-alveloar infections could be explained by differences in dental disease patterns susceptibility/resistance profile of oral bacteria. One of the main findings of the current study is that the mean number of prescriptions per dentist in the study period in Norway and Sweden is 29 and 32 prescriptions, respectively. In England and Scotland, the mean number of prescriptions per dentist in the study period is 156 and 104 prescriptions, respectively. In addition, the narrower spectrum Phenoxymethyl penicillin are more widely prescribed in Norway and Sweden whilst the broader spectrum Amoxicillin is largely prescribed in England and Scotland. The factors that influence the difference in prescription rate between dentists in these countries warranty further investigation.

This is the first study to use National data to assess antibiotic prescription volume and prescription patterns for dental infections in four northern European countries. A total of 27,076,599 prescriptions issued by dentists working in England, Scotland, Norway and Sweden over the period 2010–2016 were analysed in this study. Prescriptions for dental infections accounted on average for 6–8% of the total prescriptions in Primary care from these countries. An important caveat of this data is that in the UK it probably underestimates the actual number of antibiotic prescriptions since the data source does not capture private prescriptions, and the number of patients receiving private treatment is estimated to be around 33% in the UK [21].

All countries show a decline in antibiotic prescriptions since the start of the analysis period indicating that stewardship initiatives in these countries are having an overall downward effect on prescription volumes. From 2010 to 2016, Sweden dentists reduced their total number of prescriptions by 19% followed by England 18%, Scotland 13% and then Norway by 4%. Although the least reduction of the total number of prescriptions was in Norway, however, the DID_100,000_ and number of prescriptions per dentists in Norway are still the lowest for all antibiotics, except Clindamycin, compared to England, Scotland and Sweden. The significantly lower prescribing rates in other Northern European countries compared to the UK is a pattern observed in other Specialties [22]. Lower prescribing rates in Norway and Sweden may also be due to differences in diseases levels in addition to prescribing habits. However, the findings reported in our study have several limitations such as, the UK data only relates to NHS primary care dentistry and excludes private provision and hospital data. As with all prescribing, this data takes no account of whether the prescribing was appropriate or not and does not include dose, frequency or duration. The results and conclusions must be interpreted in the context of these limitations.

One of the more marked differences in the antibiotic prescribing patterns between the UK and Northern Scandinavian countries is the selection of Amoxicillin over Phenoxymethyl penicillin. In the UK the British National Formulary highlights either Amoxicillin (500 mg every 8 h in adults for 5 days) or Metronidazole (400 mg every 8 hourly in adults for 3–7 days) [23]. The dose and duration for Phenoxymethyl penicillin in adults is 500 mg 6 hourly for 5 days. UK professional society guidance, such as the Faculty of General Dental Practice (FGDP) and the Scottish Dental Clinical Effectiveness Programme (SDCEP) also highlights the first choice of Amoxicillin for use in acute dento-alveolar (dental abscess) infections [2, 3]. In Norway and Sweden, the recommendations for treatment choice for acute dento-alveolar infections are given in the Norwegian Directorate of Health [1] which recommends Phenoxymethyl penicillin (660 mg every 6 hourly in adults for 5 days) and in Sweden the recommended regime by the Swedish Medical Products Agency is 1,6 g Phenoxymethyl penicillin every 8 h for 5–7 days [24]. Prescription of Phenoxymethyl penicillin in Norway and Sweden as the first line of drug to treat infections in outpatients is common not only in dentistry but also in general medicine for management of upper respiratory tract infections [1]. In Norway for example, the prescription of Phenoxymethyl penicillin contributes to about 70% of the total antibiotic prescriptions in the country [18].

The narrower spectrum Phenoxymethyl penicillin are more widely prescribed in Norway and Sweden whilst the broader spectrum Amoxicillin is largely prescribed in England and Scotland. The Amoxicillin DID_100,000_ for Norway and Sweden is at least 10 times less than that of England and Scotland. Similar pattern is also found in other European countries where DID_100,000_ attributed to dental prescription of Amoxicillin in Belgium is approximately 10 times higher than that of Norway and Sweden [25]. The question arises whether the differences between Scandinavia and the UK in prescription behaviour among dentists is because of differences in susceptibility among oral bacteria or because of attitudes toward antibiotic prescribing influenced by educational curriculum, stewardship programs and national guidelines. Of further interest is the proportionally higher use of clindamycin in Norway and Sweden, in the UK one of the drivers to reduce prescribing of clindamycin is it’s link to *C. difficile* infection. It is unclear why there is a higher prescription rate for this antibiotic in Norway and Sweden.

The majority of oral penicillins are absorbed, so that they yield peak levels 1–2 h after ingestion with approximately 60 and 75% absorption following oral administration for Phenoxymethyl penicillin and Amoxicillin, respectively [26]. Penicillins are bound to proteins (usually albumin) to varying degrees ranging from 17% for Amoxicillin to 80% for Pencillin V. Only unbound drug exerts antibacterial activity, because the bound drug is not free to interact with penicillin binding proteins. However, protein binding is a reversible process and it is possible for bound protein to be desorbed and then released to interact with bacteria in tissue [26]. Penicillin’s are well distributed to most areas of the body, and their levels in areas affected by dental abscesses are sufficient in the presence of inflammation to inhibit most susceptible bacteria [27].

Phenoxymethyl penicillin and Amoxicillin belong to the time-dependent killing agents, with a target to maintain concentrations greater than the MIC for greater than or equal to 75% of the dosing interval). Phenoxymethyl penicillin, are most active against non-ß-lactamase producing Gram-positive bacteria (viridans group streptococci, anginosus group streptococci), anaerobes and selected Gram-negative cocci. Gram positive bacteria inhibited by natural pencillins in general are more susceptible to Phenoxymethyl penicillin than to semisynthetic pencillins like Amoxicillin [28, 29]. Typical MIC’s for common Gram positive streptococci from dental infections, such as viridans streptococci are 0.01 and 0.05 μg/ml for Phenoxymethyl penicillin and Amoxicillin [26]. For anginosus streptococci these are invariably sensitive to Phenoxymethyl penicillin and Amoxicillin [30]. Amoxicillin possess the same spectrum as Phenoxymethyl penicillin, plus they are more active against Gram negative cocci and members of the family Enterobacteriaceae [31]. Both agents are susceptible to a wide range of ß-lactamases.

Antibiotic resistance in microbes recovered from the acute dental abscess has been reported to be increasing (with the exception of Metronidazole) in some populations studied over the last few decades [32–35]. Care must be taken in interpretation of studies due to differences in details of the identification of isolates, selection of appropriate breakpoints and their relevance to oral infections and lack of details on MICs (for example, not all studies report MIC_90_ data). This is illustrated by reports of resistance rates for Amoxicillin ranging from 9 to 54% of common isolates from acute dental abscesses [12]. However, analysis of a number of reports does reveal a trend that the least susceptible isolates from an acute abscess are more likely to be black-pigmented Prevotella species, such as *Prevotella intermedia*. Resistance to macrolides appears to have a higher prevalence in the ‘viridans group streptococci’, anaerobic streptococci and Prevotella species [36–38]. The prevalence of resistance to lincosamides, such as clindamycin, is low. For example, Kuriyama et al. reported on 664 isolates from 163 patients and found a clindamycin MIC_90_ of 2 mg/L for 15 strains of penicillin resistant anaerobic streptococci, with all the remaining isolates (649) having a clindamycin MIC_90_ less than 0.5 mg/L. Similar low levels of clindamycin resistance have been found in the UK and elsewhere [37, 39].

Differences in the prescribing patterns of Metronidazole are also markedly different between the UK and Northern Scandinavian countries. In England and Scotland prescription of Metronidazole in accounts for approximately 52–57% of all Metronidazole prescribed in primary care [40, 41]. In Norway and Sweden, metronidazole prescription by dentists account for 19 and 16%, respectively, of the total metronidazole prescription. The driving force for this prescribing pattern is unclear, with UK guidance provided in the BNF/FGDP/SDCEP which recommends Amoxicillin as a first line agent with clindamycin or clarithromycin as a second line agent or in patients allergic to penicillins [2, 3, 23]. Metronidazole is advocated (SDCEP) as an alternative agent in penicillin allergic patients and in the first line management of necrotising gingivitis and pericoronitis. Additional guidance (SDCEP) suggests addition of metronidazole to amoxicillin in cases of severe odontogenic infection [3]. The treatment course for Metronidazole in the UK is 400 mg every 8 h for 5 days [23]. In Norway and Sweden the recommendations for prescribing metronidazole in dental infections is also 400 mg every 8 h for 5–10 days [1, 24].

The high levels of use of Metronidazole for the management of dental infections in England and Scotland is of concern in the context of potential increase in Metronidazole resistance in anaerobic bacteria populations [42]. This is compounded by poor surveillance systems in dental infections for either detection of antibiotic resistance to beta-lactams or Metronidazole.

The prescribing levels of the macrolides (clarithromycin/erythromycin) are as expected for their recommended use as second line agents in patients allergic to penicillins. Spiramycin is an antibiotic belong to the macrolide group of antibiotics. It has a spectrum against aerobic and anaerobic bacteria. This drug is prescribed by dentists in Norway and not by dentists in England, Scotland and Sweden, although Norwegian prescription guidelines do not encourage the use of this drug. The use of spiramycin as an adjunct to mechanical therapy for treatment of periodontal infections is recommended in the Norwegian Pharmaceutical Product Compendium (Felleskatalogen), which might explain the prescription of his drug in Norway.

## Conclusion

In conclusion, this study has provided an in-sight into prescribing patterns for bacterial intra-oral infections managed by general dental practitioners from England and Scotland with Norway and Sweden. All four countries demonstrated reduction in overall numbers of items dispensed with lower prescribing patterns in Norway and Sweden. Other notable differences in prescribing patterns linked to first line choice of antibiotic between these countries existed with Phenoxymethyl penicillin the preferred choice in Scandinavia. There is some logic in a more widespread adoption of the use of the narrower spectrum Phenoxymethyl penicillin over Amoxicillin in the UK as it probably has a lesser impact on the normal flora without compromising treatment efficacy. Identification of high levels of Spiramycin prescribing in Norway may serve as a trigger for this country to review the prescribing guidance for the use of this antibiotic in managing chronic periodontal disease. Further work is required to understand the high levels of metronidazole prescriptions as this element of healthcare accounts for a significant contribution of selection pressure for the emergence of metronidazole resistance.

## Data Availability

The DDD datasets used in this study are available from the corresponding author on reasonable request.

## Abbreviations

AMR: Antimicrobial resistance
YLD: Years Lived with Disability
NHS: National Health Service
NorPD: Norwegian prescription database
ATC: Anatomical Therapeutic Chemical classification system
DDD: Defined Daily Dose
DID_100,000_: DDD per 100,000 inhabitants per day
GDP: General Dental Practice
SDCEP: Scottish Dental Clinical Effectiveness Programme
g: Gram
mg: Milligram
μg: Microgram
mL: Millilitre
Amxy: Amoxicillin
Phxy: Penicillin V
Met: Metronidazole
Clin: Clindamycin
Ery: Erythromycin
Doxy: Doxycycline
Tet: Tetracycline
Oxy: Oxytetracycline
Azy: Azithromycin
Coamxy: Co-amoxiclav
